# Rapid, Portable, Multiplexed Detection of Bacterial Pathogens Directly from Clinical Sample Matrices

**DOI:** 10.3390/bios6040049

**Published:** 2016-09-23

**Authors:** Christopher R. Phaneuf, Betty Mangadu, Matthew E. Piccini, Anup K. Singh, Chung-Yan Koh

**Affiliations:** 1 Biotechnology and Bioengineering, Sandia National Laboratories, Livermore, CA 94551, USA; crphane@sandia.gov (C.R.P.); bmangad@sandia.gov (B.M.); mpcostabile@msn.com (M.E.P.); aksingh@sandia.gov (A.K.S.); 2 Currently at Cepheid, Sunnyvale, CA 94089, USA

**Keywords:** microfluidics, diagnostics, pathogen detection, point-of-care, immunoassay, centrifugal, enteric diseases

## Abstract

Enteric and diarrheal diseases are a major cause of childhood illness and death in countries with developing economies. Each year, more than half of a million children under the age of five die from these diseases. We have developed a portable, microfluidic platform capable of simultaneous, multiplexed detection of several of the bacterial pathogens that cause these diseases. This platform can perform fast, sensitive immunoassays directly from relevant, complex clinical matrices such as stool without extensive sample cleanup or preparation. Using only 1 µL of sample per assay, we demonstrate simultaneous multiplexed detection of four bacterial pathogens implicated in diarrheal and enteric diseases in less than 20 min.

## 1. Introduction

Globally, enteric and diarrheal diseases are one of the most common causes of death in children under the age of five, second only to pneumonia [1,2,3,4]. Though treatable and preventable, diarrhea is a significant problem in the developing world due to the scarcity of safe drinking water and sanitation. Studies of this problem point to a cycle of poor health and nutrition leading to susceptibility to infection by enteropathogens, leading to acute and prolonged diarrhea that exacerbates poor health and malnutrition [5]. This cycle must be interrupted by access to both well-established and proven treatments, such as oral rehydration therapy, and diagnostic tools capable of identifying causative pathogens and informing targeted treatments and preventative measures.

Although the developed world possesses significant medical infrastructure for timely and effective diagnostics, technologies appropriate for low-resource settings have been slow to emerge. For the purposes of tackling the problem of enteric and diarrheal diseases, affordable, easy-to-use, and field-deployable tools are needed to replace conventional microbiological techniques such as culture methods that require substantial time, extensive training, and specialized facilities. In addition to high sensitivity and specificity, the ideal test platform should require minimal sample preparation and be capable of handling a variety of clinical sample types, a major challenge for diagnostics developers [6,7]. Additionally, multiplexed detection is critical for diagnosing enteric diseases since co-infections are very commonplace [8]. Differential diagnostics is also important for pursuing the correct course of action; in the case of enterohemorrhagic *E. coli*, administration of antibiotics is counter-indicated, as it would cause the cells to lyse, releasing large amounts of toxin into the body.

Numerous commercial diagnostic systems have been developed with screening kits aimed at the detection of enteric infections. These include the BD Max system from Becton Dickinson (East Rutherford, NJ, USA), the PanNAT system from Micronics (Redmond, WA, USA), the FilmArray system from Biofire Diagnostics (Salt Lake City, UT, USA), and the xTag gastrointestinal pathogen panel (GPP) assay from Luminex (Austin, TX, USA) [9]. Although these devices have been demonstrated as effective diagnostic tools, most are not designed for portable, standalone operation, as required for use in a remote setting. Additionally, limitations such as the need for large sample volumes, costly reagents, and low throughput prevent these technologies from having a significant impact on global problems such as diarrheal disease.

Efforts in the field of microfluidics hold great promise for the alleviation of such global public health problems by meeting the need for reliable point-of-care diagnostics. Through miniaturization and integration of various bioanalytical processes, microfluidic technologies have the potential to offer rapid, cost-effective, and portable solutions to medical needs in the developing world [10]. Within the application space of enteric screening, a variety of microfluidic detection modalities have been reported, including electrochemical [11,12], surface plasmon resonance (SPR) [13], and molecular methods such as polymerase chain reaction (PCR) [14,15,16] and loop-mediated isothermal amplification (LAMP) [17,18,19,20,21,22,23]. A notable subset of the microfluidic system types is centrifugal, or lab-on-a-disc, devices, first developed in the late 1990s [24]. The simplicity of operation, the lack of complicated external instrumentation for sample transport (e.g., pumps), the ability to easily eliminate bubbles, and the inherent capability of density-based separation make centrifugal systems uniquely suited for diagnostics at the extreme point-of-care [25,26]. Using basic rotary control, simple microfluidic features can provide a multitude of unit operations, such as valving, metering, and mixing, making it easy to achieve scalable parallelization and integration for an array of samples [27,28,29]. Recently, several centrifugal systems designed for pathogen detection via LAMP have been reported [17,19,21]. These devices illustrate impressive sample transport control and sensitive detection but require at least 60 min runtimes and suffer from the many disadvantages of device complexity, including a high manufacturing cost, difficulty of use, and reliability issues.

We present here a portable centrifugal microfluidic system that uses a sedimentation-based immunoassay, reported in previous work by our group [30,31,32,33], to detect a panel of four pathogenic bacteria: *E. coli*, *Listeria*, *Salmonella*, and *Shigella*. The immunoassays were performed on simple, inexpensive microfluidic discs that were loaded into a compact, battery-powered instrument to perform centrifugation and subsequent detection using laser-induced fluorescence (LIF). With the ability to multiplex the detection of several pathogens of interest from the same sample, we demonstrate highly selective detection of the full panel of enteric diseases in a stool sample. In addition, we demonstrate the sensitivity for each target pathogen as well as the versatility and real-world viability of our assay by performing detections in a variety of sample matrices.

## 2. Materials and Methods

### 2.1. Microfluidic Platform Development

The prototype device utilizes an epifluorescent optical system. A 4 mW 635 nm diode laser (Edmund Optics) passes through a custom-sized 640 nm, 14 nm bandwidth excitation filter (Semrock, Rochester, NY, USA) and 676 nm, 29 nm bandwidth emission filter (Semrock). The other physical components of the optical hardware (cages, holders) were purchased from Thorlabs (Newton, NJ, USA). A photomultiplier tube (Hamamatsu, Japan) was used for detection of the fluorescence signal. A custom stepper motor (Lin Engineering, Morgan Hill, CA, USA) was used to rotate the disk and provide positional accuracy for channel registration. Control electronics consisted of a Stellaris series motherboard (Texas Instruments, Dallas, TX, USA) paired with custom auxiliary circuit boards (fabricated by ExpressPCB, Santa Barbara, CA, USA). Communication to the device was achieved via Bluetooth connection. Software was created in-house using Code Composer (Texas Instruments). Labview was used to create the graphical user interface (GUI). The device housing was designed in-house and 3D printed by ProtoMold (Maple Plain, MN, USA).

**Figure 1:**
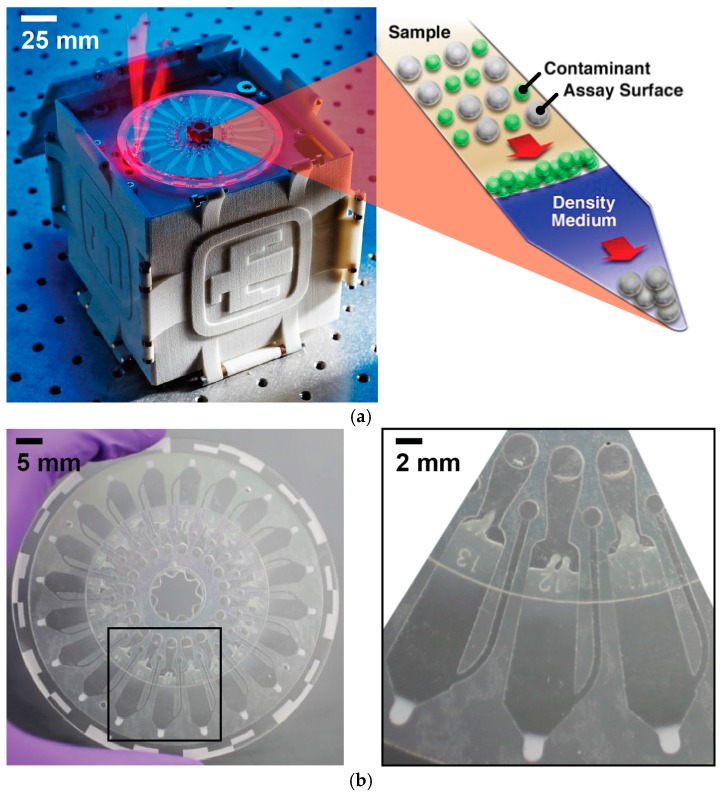
(**a**) The microfluidic platform is shown (**left**) performing epifluorescence detection (note: the lid is closed when in actual use). The principles of the sedimentation-based immunoassay (**right**) illustrate the ability to concentrate a bead-bound analyte while excluding contaminants; (**b**) The 20-channel microfluidic disc is made from lasercut PMMA and PSA layers. The close-up illustrates the channel architecture and the appearance of a used disc with the concentrated pellet of beads shown at the tip of each channel.

The device and an overview of principles of the centrifugal sedimentation assay are shown in Figure 1a. Antibody-functionalized microparticles (1 µm silica beads) and unbound fluorescently-tagged antibodies comprise a detection suspension which is mixed in equal volumes with the sample. The resultant mixture comprising the sample and detection suspension is then loaded into the channel formed by pressure sensitive adhesive in the middle layer of the device. In the channel is pre-loaded the density medium through which the microparticles will differentially sediment from objects that are less dense. The microparticles then form a pellet at the periphery of the disc due to centripetal acceleration and channel geometry, shown in Figure 1b. In the presence of the analyte of interest, the fluorescently-tagged detection antibody will form a complex with the microparticle-bound capture antibody with the antigen serving as a bridge. The fluorescence of the microparticle pellet is measured to quantify concentration of the target analyte in the sample as compared to a standard curve. The entire assay requires less than 30 min (compared to several hours for other in vitro assay approaches). Furthermore, the scale of the device allows for small samples sizes (7 µL per sample), whereas other assays typically use much larger volumes (e.g., 100 µL for ELISA).

### 2.2. Microfluidic Disc Design and Fabrication

The microfluidic disc has an outer diameter of 89 mm and is composed of three layers. The top and bottom are made from 1.5-mm-thick cast poly(methyl methacrylate) (PMMA) sheets (McMaster-Carr) that were etched and cut using a CO_2_ laser cutter (Universal Laser Systems). The middle layer is made from 75-µm-thick double-sided, pressure-sensitive adhesive (PSA) (Fralock, Valencia, CA, USA) and defined the channels. Inlet ports were 1.5 mm diameter; channel length (in the radial direction) was 25 mm and channel width (in the circumferential direction) had a maximum of 5.86 mm and narrowed to 1.38 mm at the tip. The channel design served both to isolate the individual regions of detection spatially, thereby reducing inter-channel interference, and to concentrate the resultant bead pellet physically by confinement.

### 2.3. Reagent Preparation

Carboxylic acid-functionalized silica microparticles (Bangs Labs, Fishers, IN, USA), 1 µm in diameter, were activated with an excess of *N*-ethyl-*N*′-(3-dimethylaminopropyl)carbodiimide and n-hydroxysuccinimide (0.5 mmoles of each) at pH 6.4 in 1 mL of 100 mM 3-(*N*-morpholino)propanesulfonic acid to form the succinimidyl ester. Then particles were washed once with 100 mM 3-(*N*-morpholino)propanesulfonic acid and subsequently twice with phosphate buffered saline (PBS; 138 mM NaCl, 2.7 mM KCl, 10 mM Na_2_HPO_4_, pH 7.4). The capture antibody was added to a final concentration of 0.6 g/L and the solution was raised to pH 8.15 with 1 M NaHCO_3_ and reacted at 4 °C for four hours. Any remaining activated ester was quenched with 200 mM glycine in PBS and washed in PBS three times. The particles were then twice blocked with 1% (w/v) bovine serum albumin for 30 min at 4 °C. Next, the particles were washed in wash buffer (0.08% (w/v) Tween-20, 0.02% (w/v) Pluronic F127, 0.09% (w/v) n-dodecyl β-d-maltoside, 0.8 mM NaN_3_, 0.1% (w/v) BSA, in PBS) and resuspended in wash buffer to a concentration of 12% solids. The procedure was the same for each capture antibody for each pathogen. Antibodies used were: anti-*Shigella* species (rabbit polyclonal, Kirkegaard & Perry Laboratories, cat# 01-90-01, immunogen: heat-killed whole cells), anti-*E. coli* O157:H7 (goat polyclonal, Kirkegaard & Perry Laboratories, cat# 01-95-90, immunogen: heat-killed whole cells), anti-*Listeria* species (goat polyclonal, Kirkegaard & Perry Laboratories, cat# 01-90-95, immunogen: antigens from various strains of *Listeria*), anti-*Salmonella* (goat polyclonal, Kirkegaard & Perry Laboratories, cat# 01-91-99, immunogen: heat-killed whole cells).

Detection antibodies were labeled with AlexaFluor 647 (Life Technologies, Carlsbad, CA, USA). For each pathogen, the same polyclonal antibody was used for detection. The same procedure was used to label each detection antibody. Antibodies were first buffer exchanged into PBS using desalting spin columns (7 kDa molecular weight cut-off, Pierce). Then 100 µg of activated dye was dissolved into 4 µL of dimethylsulfoxide and added to 100 µg of antibody in 176 µL of PBS. Then 20 µL of 1 M NaHCO_3_ was added to raise the pH to 8.15. The reaction proceeded at room temperature for 15 min in the dark with end-to-end rotation. After the reaction was complete, unreacted dye was separated from the antibody by another desalting column. Dye to antibody ratios were determined spectrophotometrically by UV absorbance.

### 2.4. Immunoassay Protocol

First, 1 µL of antigen and 1 µL of detection antibody (final concentration 0.75 g/L) were added to 6 µL of a 12% (w/v) suspension of capture particles (as prepared above). Antigen dilutions were generated by first diluting stock bacteria to 10^6^ bacteria/mL (lot-dependent concentrations from the manufacturer) in the matrix of interest and subsequently serially diluting in the same matrix. De-identified, pooled, whole, normal human blood (lithium heparin) from healthy adult donors, mouse serum, normal human saliva, normal human urine, and normal mouse stool were purchased from Innovative Research (Novi, MI, USA) and used without further treatment. Liquid matrices and suspensions (i.e., blood, serum, saliva, and urine) were used as the assay diluent as is. Feces were diluted in PBS to a concentration of 50% solids (w/v) prior to use to aid in pipetting. Antigens and antibodies were incubated at room temperature for 15 min. After incubation, the suspensions were mixed by pipet and 7 µL were added to the channel of the microfluidic disc above the preloaded density medium. Discs were then placed into the prototype device, secured with a thumbscrew, and the analysis protocol was started via the computer-controlled GUI. The device automatically spins the disc at 8000 RPM, indexes the channels, analyses each channel via laser-induced fluorescence, and reports relative fluorescence values to the connected computer. The fluorescence values are then exported to Prism (GraphPad Software, San Diego, CA, USA) for data analysis and reduction. Replicate data points were averaged, standard deviation was graphed as the error, and the data were fit to a four-parameter logistic curve. Limits of detection were interpolated from the curve fit using the IUPAC definition of three standard deviations above the noise.

### 2.5. ELISA Protocol

Capture antibodies were diluted to a concentration of 10 mg/L in Tris-buffered saline (TBS; 50 mM Tris, 150 mM NaCl, pH 8). To a high-binding 96-well plate, 0.1 mL of the diluted capture antibody was added to each well and allowed to incubate overnight at 4 °C. After incubation, the liquid was removed and the plates were washed three times with 0.2 mL of TBS. The plate was then blocked with 0.2 mL of blocking buffer (5 wt/vol % dry non-fat milk in TBS) for 2 h at room temperature. Blocking buffer was then aspirated and the plate was washed three times with 0.2 mL of TBS. The antigen was interest was diluted as above and 0.1 mL was added to the plate. The antigen was allowed to bind for three hours at room temperature. Samples were aspirated and the plate was washed three times with TBS with 0.05 wt/vol % Tween-20 (TBST). Diluted detection antibody (HRP-conjugated, 1:100,000 dilution in TBS, 0.1 mL) was added to the plate and allowed to incubate for two hours at room temperature. The detection antibody solution was then aspirated and the plate washed four times with TBST. To the plate, 0.1 mL of substrate (SuperSignal Femto, Thermo Fisher Scientific, Waltham, MA, USA) was added and analyzed by a plate reader (Envision, Perkin Elmer, Waltham, MA, USA).

### 2.6. Safe Handling of Human and Animal Products

All human samples were commercially acquired and screened for common blood-borne pathogens. Animal samples were likewise commercially acquired and screened. Nonetheless, universal precautions were observed while handling sample matrices including personal protective equipment, aerosol-resistant liquid handling tips, and biological safety cabinets.

### 2.7. Multiplexed Analysis

To demonstrate specificity, selectivity, and multiplexing, a pool of bacteria was created consisting of 100,000 cells/mL of each bacteria except the analyte of interest. For example, to test for specificity and selectivity of *E. coli* antibodies, a background pool consisting of *Salmonella*, *Shigella*, and *Listeria* was made with each of these bacteria at 100,000 cells/mL. *E. coli* was then titrated into this pool at various concentrations. Each immunoassay proceeded as above.

## 3. Results

**Figure 2:**
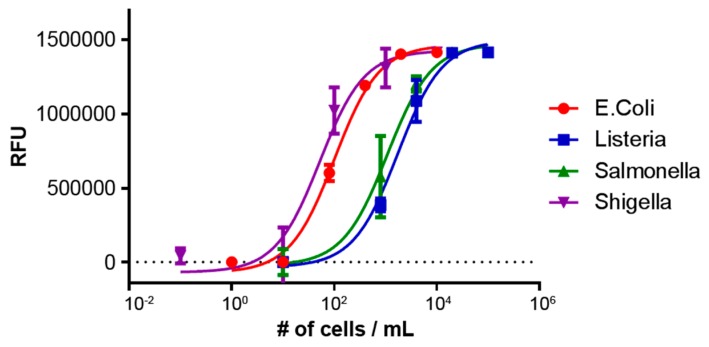
Multiplexed detection of *E. coli*, *Listeria*, *Salmonella*, and *Shigella* from a stool sample. Each target pathogen was detected in the presence of a background pool of the other non-targeted pathogen to demonstrate specificity and selectivity.

In order to demonstrate multiplexed detection of each of the pathogenic bacteria simultaneously, a single sample was screened directly from stool on a single disc. A range of bacteria concentrations were tested in this multiplexed format and the resulting fluorescence measurements, displayed in relative fluorescence units (RFU), are shown in Figure 2. Each target was successfully detected in the presence of other background pathogens and complex sample matrices, conditions that would prove difficult for traditional methods without the use of extensive sample preparation.

**Figure 3:**
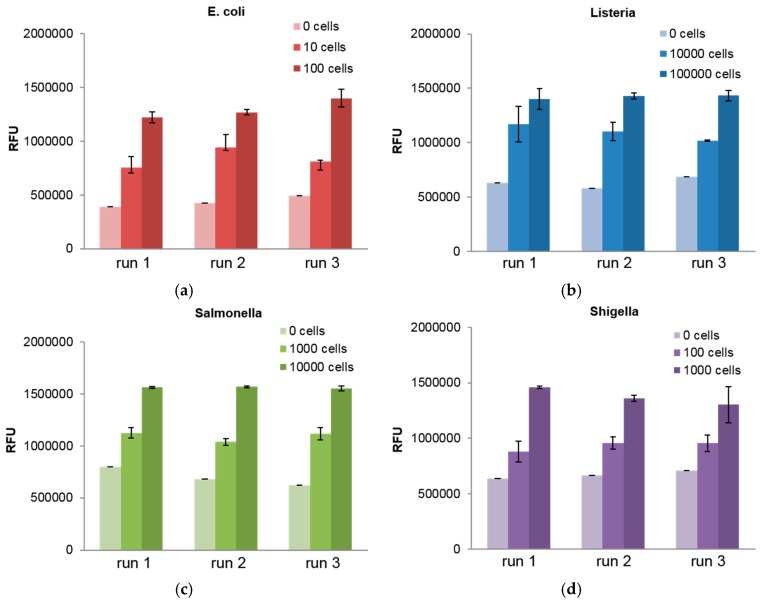
The sensitivity of our detection was demonstrated with three disc runs for a range of cell counts for all bacterial targets: (**a**) *E. coli*; (**b**) *Listeria*; (**c**) *Salmonella*; and (**d**) *Shigella*. These samples were run in assay buffer.

Furthermore, we characterized the sensitivity of the platform by screening a series of dilutions of each bacterial pathogen in variety of sample matrices. For each target, three discs were run and the results for samples in assay buffer are shown in Figure 3. From this we demonstrate limits of detection (LOD), defined by IUPAC standards to be three standard deviations above the noise, down to as low as 10 s of cells for the bacteria of interest. It can be seen that *E. coli* and *Shigella* are the most sensitively detected, following by *Salmonella* and *Listeria*. This trend agrees with the results of the multiplexed detection experiments shown in Figure 2. The full set of LOD values for each bacteria and sample matrix is presented in Table 1.

**Figure 4:**
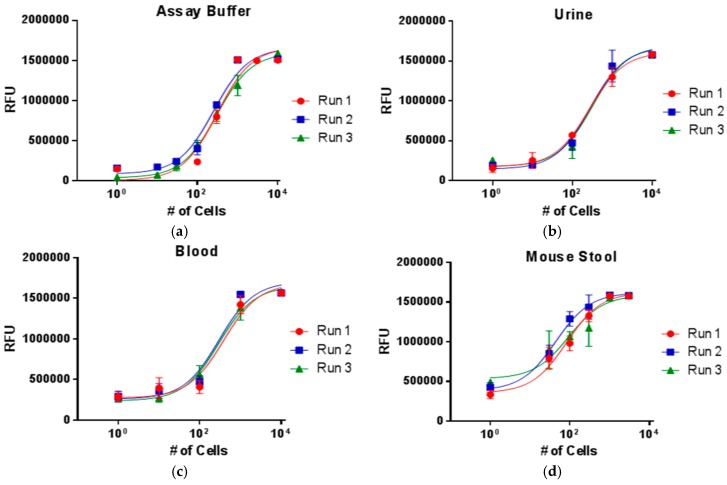
The ability to work with clinical samples without extensive sample preparation was demonstrated with the detection of *E. coli* in a variety of complex sample matrices: (**a**) buffer; (**b**) urine; (**c**) blood; and (**d**) mouse stool.

As a proof-of-concept demonstration of the platform’s versatility, we demonstrate the detection of *E. coli* from a variety of clinical sample matrices such as urine, blood, and stool for a range of cell concentrations (Figure 4). Though bacteria will typically not be found in all of these matrices, the results demonstrate a robust, linear response independent of the sample matrix. It is interesting to note that the limits of detection in biological samples may be improved compared to the assay buffer in some instances. We propose that this phenomenon may be explained by the excluded volume effect, or macromolecular crowding [34,35]. The biological sample matrices are highly complex with a large diversity and concentration of molecules. Therefore, a larger number of molecules will interact with the antibodies on the surface of the particles. Weakly binding species will be further pushed off of the beads by tighter binding partners and competition may decrease the amount of nonspecific binding to the particles prior to the wash in the density medium due to Stokes’ flow. The cumulative effect may be an improvement in the limit of detection by increasing specificity.

**Figure 5:**
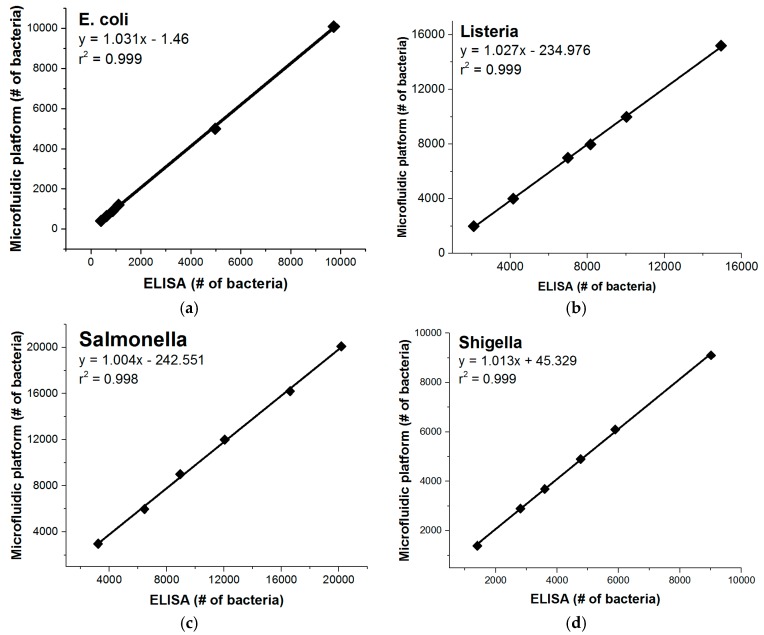
Method comparisons of the microfluidic platform to conventional ELISA detections were performed in assay buffer for (**a**) *E. coli*; (**b**) *Listeria*; (**c**) *Salmonella*; and (**d**) *Shigella*. The two methods are shown to correlate closely with neither method significantly under- or over-estimating the number of bacteria.

In order to compare the centrifugal microfluidic platform described here to gold standard techniques, we conducted a side-by-side comparison with microtiter plate ELISA. The data of these control experiments are shown in Figure 5. Similar limits of detection between the two methods are seen and the method comparison plots show good linearity between the two platforms.

## 4. Conclusions

We have demonstrated a microfluidic platform and screening methodology well suited to addressing global health issues, including the burden of enteric and diarrheal diseases on the developing world. This platform offers an affordable and field-deployable solution with high sensitivity, specificity, and selectivity. Another key advantage of the system is the ability to detect the analyte of interest directly from a clinically relevant sample matrix without extensive prior sample clean-up or preparation. Traditional microplate ELISAs rely on extensive washing steps and long incubations to achieve comparable sensitivity. The platform automates, simplifies, and expedites these steps through the use of differential sedimentation; Stokes flow provides several hundred bead-volumes of washes. The ease of use, low cost, and simplicity of the device make a compelling case for use in triage situations, disease surveillance, outbreak response and tracking, or other diagnostic needs in resource-constrained environments. Future directions for the development and application of the microfluidic platform include the addition of temperature control for conducting nucleic acid–based detection and expanding the diagnostic versatility.

## Figures and Tables

**Table 1:** The limit of detection was determined for the full panel of bacteria in a variety of sample matrices, including assay buffer, urine, blood, and stool. Singleplex detections were performed for all matrices and multiplex detections were confined to assay buffer and stool. Conventional ELISA detections in assay buffer are also shown for comparison.

	LOD (# of Cells)						
	*Microfluidic Singleplex*				*Microfluidic Multiplex*		*Conventional Singleplex*
	Buffer	Urine	Blood	Stool	Buffer	Stool	Buffer
*E. coli*	11	34	33	9	51	31	38
*Listeria*	999	796	1668	320	2849	238	1745
*Salmonella*	2416	703	1200	974	1154	328	2648
*Shigella*	53	33	61	20	94	12	1236
